# Protein kinase CK2α is overexpressed in colorectal cancer and modulates cell proliferation and invasion via regulating EMT-related genes

**DOI:** 10.1186/1479-5876-9-97

**Published:** 2011-06-25

**Authors:** Jinjin Zou, Hesan Luo, Qin Zeng, Zhongyi Dong, Dehua Wu, Li Liu

**Affiliations:** 1 Department of Radiation Oncology, Nanfang Hospital, Southern Medical University, Guangzhou 510515, Guangdong Province, China; 2 Hepatology Unit and Department of Infectious Diseases, Nanfang Hospital, Southern Medical University, Guangzhou 510515, Guangdong Province, China

## Abstract

**Background:**

Protein kinase CK2 is a highly conserved, ubiquitous protein serine/threonine kinase that phosphorylates many substrates and has a global role in numerous biological and pathological processes. Overexpression of the protein kinase CK2α subunit (CK2α) has been associated with the malignant transformation of several tissues, with not nearly as much focus on the role of CK2α in colorectal cancer (CRC). The aims of this study are to investigate the function and regulatory mechanism of CK2α in CRC development.

**Methods:**

Expression levels of CK2α were analyzed in 144 patients (104 with CRC and 40 with colorectal adenoma) by immunohistochemistry. Proliferation, senescence, motility and invasion assays as well as immunofluorescence staining and western blots were performed to assess the effect of CK2α in CRC.

**Results:**

The immunohistochemical expression of nuclear CK2α was stronger in tumor tissues than in adenomas and normal colorectal tissues. Suppression of CK2α by small-interfering RNA or the CK2α activity inhibitor emodin inhibited proliferation of CRC cells, caused G0/G1 phase arrest, induced cell senescence, elevated the expression of p53/p21 and decreased the expression of C-myc. We also found that knockdown of CK2α suppressed cell motility and invasion. Significantly, CK2α inhibition resulted in β-catenin transactivation, decreased the expression levels of vimentin and the transcription factors snail1 and smad2/3, and increased the expression of E-cadherin, suggesting that CK2α regulates the epithelial-mesenchymal transition (EMT) process in cancer cells.

**Conclusions:**

Our results indicate that CK2α plays an essential role in the development of CRC, and inhibition of CK2α may serve as a promising therapeutic strategy for human CRC.

## Introduction

Colorectal cancer (CRC) is the second-most common cause of cancer death in the West [1] and its incidence in China has increased rapidly during the past few decades [2]. Colorectal cancers can be divided into tumors exhibiting chromosomal instability and tumors exhibiting microsatellite instability [3,4]. In the last few years, molecular biology advances have led to a growing knowledge of the mechanisms underlying CRC development, including the mutational activation of oncogenes and alteration of several tumor suppressor genes, such as adenomatous polyposis coli (APC), deleted in colorectal cancer (DCC) and p53 [5-8]. However, molecular markers that indicate the occurrence and development of CRC are still needed.

Protein kinase CK2 (formerly casein kinase II) has traditionally been classified as a messenger-independent protein serine/threonine kinase that is typically found in tetrameric complexes consisting of two catalytic (α and/or α') subunits and two regulatory β subunits [9]. To date, more than 300 CK2 substrates have been identified; one third of these are implicated in gene expression and protein synthesis as translational elements [10]. CK2α-knockout mice are not viable because of defects in heart and neural tube development [11]. The disruption of CK2α expression in *Saccharomyces cerevisiae *and knockout of CK2β in mice are lethal events, indicating the importance of CK2 in the maintenance of cell viability during the normal cell life and embryogenesis [12,13]. CK2α also participates in the regulation of various cell cycle stages, presumably through phosphorylation of the proteins associated with cell cycle progression [14]. Furthermore, CK2 involvement has been found in chromatin remodeling as well as protein transcription, translation, and degradation [15-17]. Recent studies suggest that CK2 creates an environment that is favorable for the development of the tumor phenotype [18].

In the present study, we assessed CK2α expression in colorectal cancer, adenoma, and normal colorectal epithelium and found CK2α involvement in CRC tumorigenesis. Moreover, the role of CK2α in cell proliferation, senescence, motility and invasion was examined in CRC cell lines that were subjected to CK2α knockdown or to the CK2α activity inhibitor emodin. Further analysis was conducted to elucidate the mechanisms of CK2α involvement in the occurrence and development of CRC.

## Materials and methods

### Patient characteristics

We obtained paraffin-embedded samples of 104 CRCs and 40 adenomas that were diagnosed on the basis of histological and clinical findings at the Nanfang Hospital between 2005 and 2007. Prior patient consent and approval from the Institute Research Ethics Committee were obtained before we used these clinical materials for research purposes. The CRC stage was defined according to the AJCC classification. The clinical characteristics of the patients with CRC are summarized in detail in Table 1. The tumors taken from the adenoma group (20 males and 20 females; age, 28 - 73 years [mean: 50.5]) consisted of 3 serrate adenomas, 22 canalicular adenomas, 9 villous adenomas, and 6 tubulovillous adenomas.

**Table 1:** Clinicopathological characteristics of the 104 patients and expression of CK2α in CRC.

	N (%)
**Gender**	
Male	56 (53.8)
Female	48 (46.2)
**Age**	
≥55	54 (51.9)
<55	50 (48.1)
**Tumor location**	
Colon	53 (51.0)
Rectum	51 (49.0)
**T stage**	
T1-T2	49 (47.1)
T3-T4	55 (52.9)
**N stage**	
Nx-0	55 (52.9)
N1-2	49 (47.1)
**M stage**	
M0	60 (57.7)
M1	44 (42.3)
**TNM stage**	
I-II	30 (28.8)
III-IV	74 (71.2)
**Degree of differentiation**	
Well	35 (33.7)
Moderately	45 (43.3)
Poorly	24 (23.0)
**Expression of CK2α**	
Low expression	43 (41.3)
High expression	61 (58.7)

### Immunohistochemistry

Immunohistochemical staining was performed using a Dako Envision System (Dako, Carpinteria, CA, USA) following the manufacturer's recommended protocol. Briefly, all paraffin sections, 4 μm in thickness, were heated for 1 h at 65°C, deparaffinized with xylene, rehydrated through a graded series of ethanol/distilled water concentrations, submerged in EDTA buffer (pH 8.0), heated in a microwave for antigen retrieval, treated with 0.3% H_2_O_2 _for 15 min to block the endogenous peroxidase, incubated overnight with rabbit monoclonal anti-CK2α antibody (1:50; Abcam, Cambridge, UK) at 4°C, washed, incubated with horseradish peroxidase (HRP) at 4°C for 30 min, and visualized with diaminobenzidine (DAB). For negative controls, the antibody was replaced by normal goat serum.

### Evaluation of staining

The immunohistochemically stained tissue sections were scored separately by two pathologists who were blinded to the clinical parameters. For assessment of CK2α, the entire tissue section was scanned before assigning the scores. The staining intensity was scored as 0 (negative), 1 (weak), 2 (medium), or 3 (strong). The extent of staining was scored as 0 (0%), 1 (1 - 25%), 2 (26 - 50%), 3 (51 - 75%), or 4 (76 - 100%), according to the percentages of the positive staining areas relative to the entire carcinoma-involved area or, for the normal samples, the entire section. The sum of the intensity and extent scores was used as the final CK2α staining score (0 - 7). This relatively simple, reproducible scoring method gives highly concordant results between independent evaluators and has been used in previous studies [19,20]. For the purpose of statistical evaluation, tumors with a final staining score of ≥3 were considered to be positive for CK2α.

### Cell lines and culture conditions

The human colorectal cancer cell lines LoVo, SW480, HT29, HCT116 and LS174T were maintained in RPMI 1640 (Gibco, Grand Island, NY, USA) supplemented with 10% fetal bovine serum at 37°C in a 5% CO_2 _humidified incubator.

### CK2α siRNA

Cells were seeded onto a six-well plate 16 h before transfection. In each well, 100 pmol of CK2α siRNA (CSNK2A1 siRNA: 5'-GAUGACUACCAGCUGGUUC-3') or scramble sequences and 5 μl of Lipofectamine 2000 (Invitrogen, Carlsbad, CA, USA) were added to Opti-MEM medium and mixed gently. The plate was incubated for 48 h until it was ready for further assay.

### Western blot analysis

Cells and tissues were washed twice with cold phosphate-buffered saline (PBS) and lysed on ice in RIPA buffer (1 × PBS, 1% NP40, 0.1% SDS, 5 mM EDTA, 0.5% sodium deoxycholate, and 1 mM sodium orthovanadate) with protease inhibitors. Whole extracts were resolved on 10% SDS polyacrylamide gels and electrotransferred to polyvinylidene fluoride (PVDF; Immobilon P; Millipore, Bedford, MA, USA) membranes, which were then blocked in 5% non-fat dry milk in Tris-buffered saline (TBST) (pH 7.5; 100 mM NaCl, 50 mM Tris, and 0.1% Tween-20) and immunoblotted with rabbit anti-CK2α monoclonal antibody (1:800; Abcam), mouse anti-E-cadherin (1:500; Santa Cruz Biotechnology, Santa Cruz, CA, USA), anti-β-catenin (1:500; Santa Cruz), mouse anti-vimentin (1:500; Santa Cruz), mouse anti-C-myc (1:200; Santa Cruz), mouse anti-p53 (1:200; Santa Cruz), mouse anti-p21 (1:200; Santa Cruz), mouse anti-GAPDH monoclonal antibody (1:1000; Santa Cruz), rabbit anti-snail1 (1:750; Bioworld Technology, St. Louis Park, MN, USA), or rabbit anti-smad2/3 (1:750; Cell Signaling Technology, Beverly, MA, USA) overnight at 4°C, followed by their respective secondary antibodies conjugated to horseradish peroxidase (HRP). The signals were detected by enhanced chemiluminescence (ECL; Pierce, Rockford, IL, USA). The images were analyzed by Image J software.

### Immunofluorescence staining

Cells were cultured on coverslips overnight, fixed with 4% paraformaldehyde for 20 min, treated with 0.25% Triton X-100 for 10 min, blocked in 10% normal blocking serum at room temperature for 10 min, incubated with mouse monoclonal anti-β-catenin (1:50; Santa Cruz) at 4°C overnight, washed with PBS three times, incubated with TRITC (teramethylrhodamine-6-thiocarbamoyl)-conjugated anti-mouse secondary antibodies (Invitrogen, Carlsbad, CA, USA) for 30 min at room temperature, and stained with 4,6-diamidino-2-phenylindole (DAPI; Invitrogen).

### In vitro cell growth assay

The cells were prepared at a concentration of 1 × 10^4 ^cells/ml. Aliquots (100 μl) were dispensed into 96-well microtiter plates. The cells were incubated for 1, 2, 3, 4, 5, or 6 days, and the 3-(4,5-dimethylthiazol-2-yl)-2,5-diphenyltetrazolium bromide (MTT) assay was performed by adding 20 μl of MTT (5 mg/ml; Promega, Madison, WI, USA) for 4 hours. When the MTT incubation was complete, the supernatants were removed. Dimethyl sulfoxide (Sigma, St. Louis, MO, USA) was added to each well (150 μl). Fifteen minutes later, the absorbance (OD) of each well was measured with a microplate reader set at 570 nm.

### Colony formation assay

Approximately 1 × 10^2 ^cells from each treatment group were seeded in triplicate wells (3 cm in diameter) of a six-well culture plate, incubated at 37°C for 12 days, washed twice with PBS, and stained with Giemsa solution. The number of colonies containing more than 50 cells was counted under a microscope.

### Senescence-associated β-galactosidase staining

Cells were seeded in triplicate on 12-well plates, fixed with 4% paraformaldehyde for 30 min, and stained with senescence-associated β-galactosidase (SA-β-gal) solution (Invitrogen). The numbers of blue-stained (SA-β-gal-positive) and total cells were manually counted under a microscope and averaged for three regions per sample well. The percentage of SA-β-gal-positive cells was calculated accordingly.

### Flow cytometry assay

Cells were harvested at an exponential growth phase, and single-cell suspensions containing 1 × 10^6 ^cells were fixed with 70% alcohol. The cell cycle was monitored using propidium iodide (PI) staining of nuclei. The fluorescence of DNA-bound PI in cells was measured with a FACScan flow cytometer (BD Biosciences), and the results were analyzed with ModFit 3.0 software (Verity Software House, Topsham, ME).

### Wound migration assay

Monolayers were wounded by scraping with a 200-μl pipette tip. Scratches were monitored for the percentage of wound closure over the next 24 h. The wound was measured in 12 places located at preset distances and averaged. Wound healing was quantified, and statistical analysis was conducted relative to the control siRNA.

### Tumor cell invasion assay

Warm serum-free medium was added to the top chamber of the cell invasion chamber (Chemicon, Temecula, CA, USA) to rehydrate the ECM layer for 2 h at room temperature. Tumor cells in serum-free medium (300 μl containing 1 × 10^5 ^cells) were added to the top chamber. The bottom chamber was prepared with 10% FBS as a chemoattractant. After 18 h of incubation, noninvasive cells were removed with a cotton swab. The cells that had migrated through and adhered to the lower surface of the membrane were fixed with methanol, stained with hematoxylin, and counted under a microscope in five randomly selected fields at × 200 magnifications.

### Statistical analysis

All statistical analyses were carried out using the SPSS statistical software package, version 13.0 (SPSS, Chicago, IL, USA). A chi-squared test was used to analyze the differential expression of CK2α in colorectal cancers, adenomas and adjacent normal colorectal mucosa. The Mann-Whitney *U*-test and Kruskal-Wallis *H*-test were used to analyze the relationship between CK2α expression and gender, age, tumor location, degree of differentiation, T stage, N stage, M stage, and clinical stage. Paired *t*-tests, Student's *t*-tests, factorial analysis and one-way ANOVA were used to analyze the findings of the *in vitro *cell assay. A *P *value of less than 0.05 was considered statistically significant.

## Results

### CK2α is overexpressed in colorectal cancer

**Figure 1:**
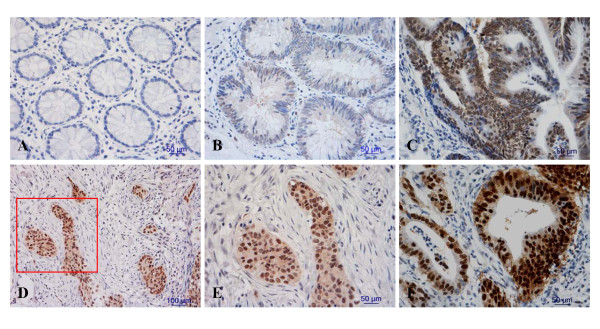
**Immunohistochemical detection of CK2α expression in colorectal cancers, adenomas and adjacent normal colorectal mucosa**. Staining was (A) negative in normal colorectal epithelium cells, (B, C) weak to moderate in the nuclei of colorectal adenoma cells, (D, E, F) and strong in the nuclei of colorectal cancer cells. (E is a close-up of the inset in D [framed in red]). Original magnification: × 200 (D), × 400 (A, B, C, E, F).

**Figure 2:**
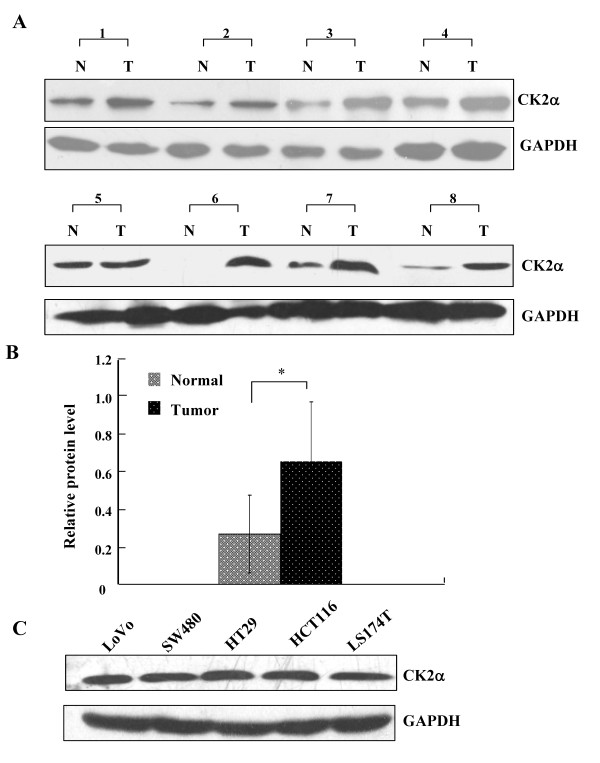
**CK2α protein expression in CRC tissues and cell lines**. (A) Western blot analysis of CK2α expression in eight pairs of CRC tissues and adjacent, normal colorectal mucosa tissues. N: normal colorectal mucosa tissue; T: tumor tissue. (B) Quantitative analysis of CK2α protein expression in eight pairs of CRC tissues and adjacent normal colorectal mucosa tissues. *Columns*, mean CK2α protein level after normalizing the data to GAPDH expression; *bars*, SD. *P < 0.01. (C) Western blot was used to detect CK2α expression in five CRC cell lines. GAPDH expression was used as a loading control.

CK2α protein expression was analyzed in 144 patients (104 with CRC and 40 with colorectal adenoma). Staining for CK2α was nearly negative in all of the normal colorectal epithelium samples (Figure 1A), and nuclear staining for CK2α was extremely weak in only 11 normal colorectal epithelium samples (11 of 86, 12.8%), positive in 17 of 40 (42.5%) colorectal adenoma samples (Figure 1B, C), and positive in 61 of 104 (58.7%) CRC samples (Figure 1D, E, F). CK2α immunoexpression was much stronger in CRC than in adenomas, while its expression was greater in adenomas than in normal colorectal epithelium (χ^2 ^= 42.035, *P *< 0.05). These data indicate that CK2α may have a role in the process of CRC tumorigenesis. We also assessed CK2α expression in 8 normal-CRC tissue pairs by western blot. Similar to the result in our immunohistochemistry assay, CK2α expression was significantly higher in colorectal tumor tissues than in normal colorectal tissues (Figure 2A, B) (*P *< 0.01). In addition, CK2α was expressed in five CRC cell lines (Figure 2C).

### CK2α overexpression is correlated with T classification in colorectal cancer

Next, we investigated the association between CK2α expression and the clinicopathological characteristics of CRC cases and found that CK2α overexpression was significantly associated with T classification (*P *= 0.002). The expression of the CK2α protein in CRC in the T3-T4 stage was significantly higher than in the T1-T2 stage. However, no significant correlation was found between CK2α expression and gender, age, degree of differentiation, N classification, distant metastasis, or location (Table 2) (*P *> 0.05). Because T describes how far the main (primary) tumor has grown into the wall of the intestine and whether it has grown into nearby areas, we speculated that CK2α may participate in CRC cell invasion.

**Table 2:** Correlation between the clinicopathological features and expression of the CK2α protein.

		CK2α (%)		
			
Characteristics	N	Low expression	High expression	*P*
**Gender**				0.646
Male	56	22 (39.3)	34 (60.7)	
Female	48	21 (43.8)	27 (56.2)	
**Age**				0.897
≥55 y	54	22 (40.7)	32 (59.3)	
<55 y	50	21 (42.0)	29 (58.0)	
**Tumor location**				0.554
Colon	53	21 (39.6)	32 (60.4)	
Rectum	51	22 (43.1)	29 (56.9)	
**T stage**				0.002*
T1-T2	49	21 (42.9)	28 (57.1)	
T3-T4	55	15 (27.2)	40 (72.7)	
**N stage**				0.515
Nx-0	55	20 (36.4)	35 (63.6)	
N1-2	49	23 (46.9)	26 (53.1)	
**M stage**				0.632
M0	60	26 (43.3)	34 (56.7)	
M1	44	17 (38.6)	27 (61.4)	
**TNM stage**				0.539
I-II	30	11 (36.7)	19 (63.7)	
III-IV	74	32 (43.2)	42 (56.8)	
**Degree of differentiation**				0.632
Well	35	13 (37.1)	22 (62.9)	
Moderately	45	21 (46.7)	24 (53.3)	
Poorly	24	9 (37.5)	15 (62.5)	

*Statistically significant difference.

### CK2α regulates growth, proliferation and senescence of CRC cell lines

**Figure 3:**
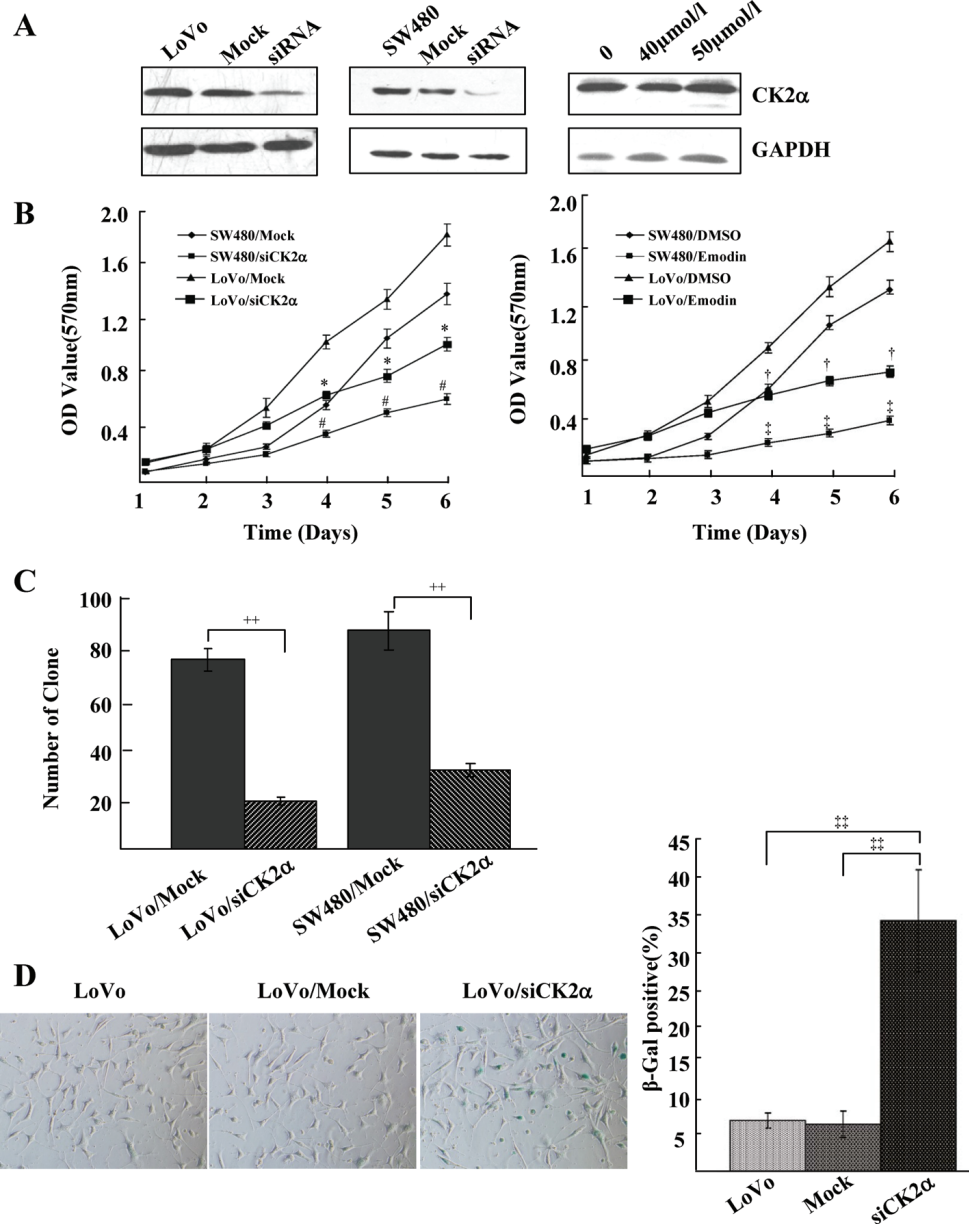
**Knockdown of CK2α inhibited cell proliferation and promoted cell senescence of CRC cell lines**. (A) Western blot analysis of CK2α protein in lysates of cells transfected with a specific CK2α siRNA or treated with emodin. GAPDH expression was used as a loading control. (B) MTT assay of the proliferating cells transfected with a CK2α-specific siRNA or a nonspecific siRNA and treated with emodin. *Points*, mean of three independent experiments; *bars*, SD. *P < 0.01 versus LoVo/Mock; #*P *< 0.01 versus SW480/Mock; †*P *< 0.01 versus LoVo/DMSO; ‡*P *< 0.01 versus SW480/DMSO. (C) The number of colonies formed from cells transfected with CK2α siRNA. Colonies were stained with crystal violet and counted. *Columns*, mean of three independent experiments; *bars*, SD. ++*P *< 0.01. (D) The number of SA-β-gal-positive cells (green) 48 h after transfection with CK2α siRNA. Cells were stained with SA-β-gal staining solution. *Columns*, mean of three independent experiments; *bars*, SD. ‡‡*P *< 0.01.

Because the process of tumorigenesis is closely correlated with eternal proliferation of tumor cells, we determined whether CK2α expression plays a role in human CRC cell growth and proliferation using siRNA to knock down CK2α expression or emodin to inhibit CK2α activity (Figure 3A). The MTT assay showed that knockdown of CK2α significantly decreased CRC cell proliferation compared to the control (nonspecific siRNA) (*F *= 32.854, *P *< 0.01 for LoVo cells; *F *= 32.655, *P *< 0.01 for SW480 cells), and treatment with emodin markedly reduced proliferation (*F *= 33.290, *P *< 0.01 for LoVo cells; *F *= 57.052, *P *< 0.01 for SW480 cells; Figure 3B). Furthermore, in the colony formation assay, inhibition of CK2α expression dramatically decreased the number of CRC colonies (*t *= 20.252, *P *< 0.01 for LoVo cells; *t *= 12.034, *P *< 0.01 for SW480 cells; Figure 3C) and promoted CRC cell senescence (*t *= 43.052, *P *< 0.01; Figure 3D). Taken together, the results indicate that CK2α plays a very important role in human CRC cell proliferation and senescence. CK2α knockdown or depression visibly inhibited cell proliferation and promoted cell senescence.

**Figure 4:**
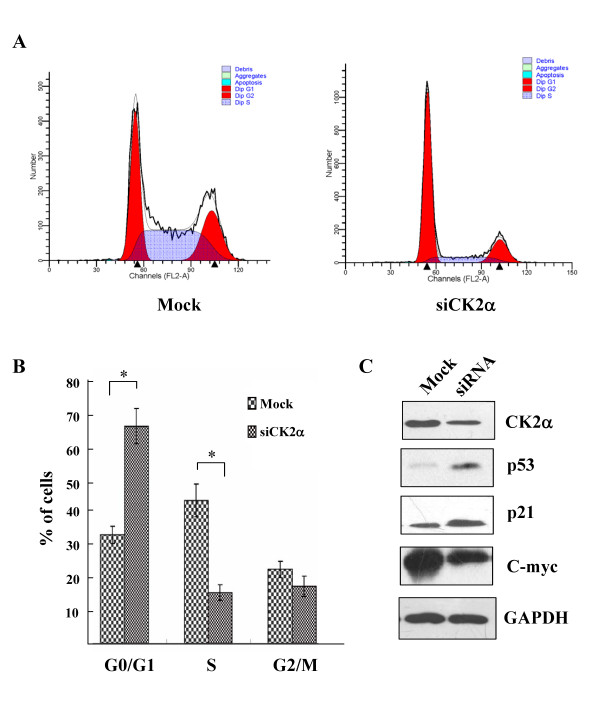
**CK2α inhibition induced G0/G1 phase arrest**. (A) LoVo cells were transfected with CK2α-specific siRNA or nonspecific siRNA, stained with propidium iodide (PI), and monitored by flow cytometry to determine the cell cycle phase distribution. (B) Comparison of the percentage of cells in each phase of the cell cycle between LoVo cells transfected with CK2α-specific siRNA and nonspecific siRNA. *Columns*, mean of three independent experiments; *bars*, SD. *P < 0.01. (C) CK2α, p53, p21, C-myc and GAPDH expression in cells transfected with CK2α-specific siRNA was detected by western blot analysis.

After CK2α knockdown, the percentage of G0/G1 phase cells significantly increased (*t *= -9.577, *P *< 0.01), and the percent of S phase cells significantly decreased (*t *= 8.749, *P *< 0.01; Figure 4A, B), indicating that CK2α knockdown induced G0/G1 phase arrest. Moreover, CK2α knockdown increased endogenous p53 and p21 expression and decreased endogenous C-myc expression (Figure 4C). Thus, it can be inferred that the inhibition of cell proliferation and cell cycle arrest in CK2α knockdown cells are associated with alterations in p53, p21 and C-myc expression.

### CK2α knockdown inhibits cell migration and invasion

**Figure 5:**
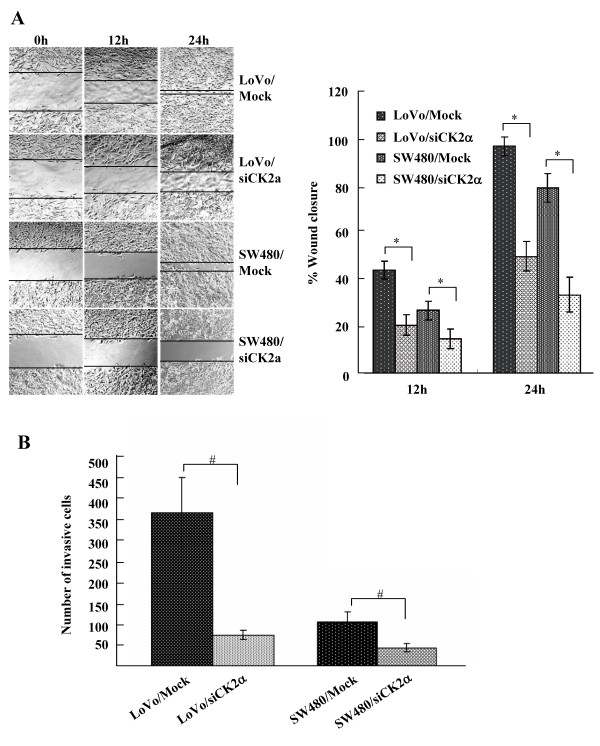
**Knockdown of CK2α inhibited cell migration and invasion of CRC cell lines**. (A) Monolayers of cells transfected with CK2α-specific siRNAs were wounded by scraping, and wound closure was followed at 0, 12, and 24 h. The distance of the wound was measured. *Columns*, mean of three independent experiments; *bars*, SD. *P < 0.01. (B) After transfection with CK2α-specific siRNAs for 18 h, cells that migrated through the filters were counted in five randomly selected fields. *Columns*, mean of three independent experiments; *bars*, SD. #*P *< 0.05.

Migration and matrigel invasion assays were performed to examine the effect of CK2α on tumor cell migration and invasion, respectively. Knockdown of CK2α greatly inhibited wound closure (*F *= 53.517, *P *< 0.01 for LoVo cells; *F *= 40.319, *P *< 0.01 for SW480 cells; Figure 5A) and invasion (*t *= 5.955, *P *< 0.01 for LoVo cells; *t *= 4.339, *P *< 0.05 for SW480 cells; Figure 5B). Accordingly, CK2α was positively correlated with CRC cell migration and invasion ability.

### CK2α knockdown reversed nuclear translocation of β-catenin and altered the expression of E-cadherin and vimentin, in association with repression of the transcription factors snail1 and smad2/3 expression

**Figure 6:**
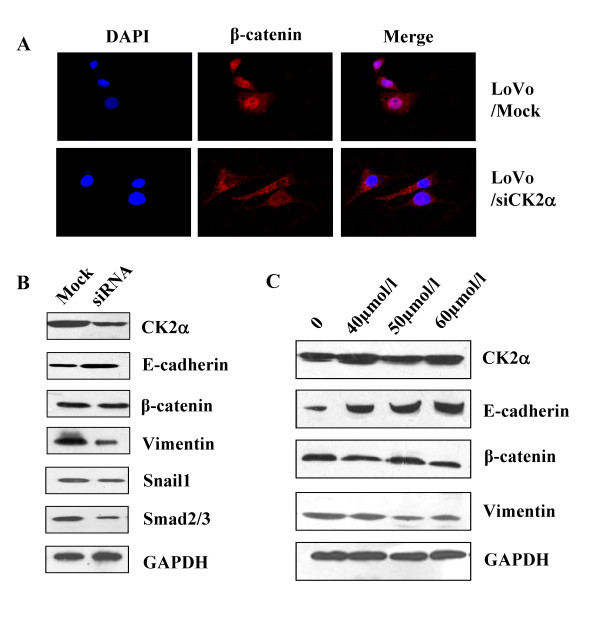
**Inhibition of CK2α reversed the nuclear translocation of β-catenin and altered EMT-related genes expression**. Reversal of EGF-induced nuclear translocation of β-catenin occurred in LoVo cells transfected with CK2α-specific siRNA (A), treated with EGF (100 ng/ml) for 2 h, and stained for immunofluorescence with β-catenin antibody (red) and DAPI (blue). (B) Western blot was used to detect the expression levels of CK2α, E-cadherin, β-catenin, vimentin and the transcription factors snail1 and smad2/3 in cells transfected with CK2α-specific siRNA. (C) One week later, in LoVo cells treated with emodin (40 μmol/l, 50 μmol/l and 60 μmol/l), the expressions of E-cadherin, β-catenin and vimentin were detected by western blot analysis. GAPDH expression was used as a loading control.

Knockdown of CK2α reversed the cytoplasmic-to-nuclear transfer of β-catenin resulted by EGF stimuli (Figure 6A). We also measured the expression levels of EMT-related genes by analyzing western blots. Cells transfected with CK2α siRNA had dramatically reduced levels of endogenous CK2α and increased levels of E-cadherin, an epithelial marker; there was no effect on the β-catenin expression level and a decreased level of vimentin, a mesenchymal marker. In addition, knockdown of CK2α decreased the expression of the transcription factors snail1 and smad2/3 (Figure 6B). The results show that CK2α knockdown represses EMT in CRC. We also treated cells with emodin and found that CK2α activity, but not protein expression, was affected. Emodin increased the expression of E-cadherin, had no effect on the expression of β-catenin, and decreased the expression of vimentin in a concentration-dependent manner (Figure 6C). Thus, depression of CK2α activity can inhibit the expression of EMT-related genes, suggesting that an increase in CK2α protein or activity may facilitate EMT and thus plays an important role in colorectal cancer invasion.

## Discussion

In this present study, we assessed CK2α expression in colorectal cancer, adenoma and normal colorectal epithelium and found that CK2α was overexpressed in CRC. Consistent with a recent study by Lin et al. [21], our findings convincingly demonstrate that CK2α was significantly upregulated in CRC. Our study further showed that CK2α protein expression levels were increased in both CRC and colorectal adenoma, and CK2α expression was much higher in CRC than in adenoma, suggesting that CK2α may be involved in the progression from adenoma to CRC. In addition, we found that CK2α overexpression was only associated with T classification, but there were no significant correlations with other clinical characteristics, possibly due to our relatively small sample size.

Several studies have shown that the dysregulation of CK2 enhances tumor cell survival [22,23], but the function of CK2α in CRC is less well known. In our study, we assessed the role of CK2α in the biological behavior of CRC. As in a recent study [21], we found that CK2α knockdown inhibited cell proliferation and colon formation in other CRC cell lines. Moreover, for the first time, we observed that, in CRC, CK2α knockdown induces G0/G1 phase arrest and promotes cell senescence. Similarly, inhibition of CK2α activity by emodin induced proliferation repression. In addition, CK2α knockdown increased p53/p21 expression and decreased C-myc expression. Accordingly, our results demonstrate that CK2α has multiple roles in the biological behavior of CRC, which is mediated by the regulation of oncogenes and anti-oncogenes, including C-myc, p53 and p21.

In our study, CK2α was found to have an important role in the biological behavior of CRC. Therefore, it is vitally important to investigate the potential regulatory mechanisms of CK2α. However, the regulatory mechanism of CK2α in contributing to the development of CRC is still unknown. The progression from normal intestinal mucosa to adenoma (adenomatous mucosa) and finally to adenocarcinoma in CRC is closely correlated with the EMT process and changes in the expression of a series of genes, such as E-cadherin, vimentin, and β-catenin [24,25]. Thus, we further investigated whether CK2α expression is associated with the EMT process. Interestingly, in our study, assays of EMT-related markers found that CK2α knockdown or activity inhibition can alter the expression of E-cadherin and vimentin and reverse the EGF-induced cytoplasmic-to-nuclear translocation of β-catenin. We confirmed that CK2α modulates the process of EMT, thereby affecting the regulation of cell migration and invasion by colorectal cancer cells. Snail1 and Smad2/3 are important transcriptional regulators of EMT that repress E-cadherin expression through binding to E-box motifs (5'-CANNTG-3') in the promoter [26-28]. In our study, we found that CK2α knockdown decreases the expressions of snail1 and smad2/3. It is clearly shown that downregulation of snail1 and smad2/3 by CK2α knockdown facilitates an increase in E-cadherin expression and EMT repression. Previous studies found that, in Her-2/neu-driven mammary tumor cells, CK2 may be involved in EMT repression, which can be induced by green tea polyphenol epigallocatechin-3-gallate (EGCG) [29]. In untransformed mammary epithelial cells, ectopic expression of CK2α facilitates the induction of EMT-related genes expression, such as that of Slug and AhR, which may thus promote the process of EMT [30]. Here we show for the first time that, in CRC, CK2α modulates the EMT process through regulating the location or expression of EMT-related genes. Recent studies have indicated that, in breast cancer, p53/p21 and C-myc not only regulate growth and senescence but are also involved in regulating the EMT process [31-34]. Thus, we inferred that, in CRC, alteration of p53/p21 and C-myc expression by CK2α knockdown may facilitate the EMT repression observed in our study. These findings may account in part for the association of CK2α overexpression with EMT in colorectal cancer. Additional studies are required to clarify the involvement of CK2α in EMT and the development of colorectal cancer.

## Conclusions

Our study demonstrates that CK2α is overexpressed in CRC and that CK2α expression is much greater in CRC than in adenoma and is greater in adenoma than in normal colorectal epithelium. Moreover, it is noteworthy to observe that, for the first time, overexpression of CK2α seems to be involved in the carcinogenesis and development of CRC through regulation of EMT-related genes. CK2α may be a promising molecular target for the diagnosis and treatment of human CRC.

## Competing interests

The authors declare that they have no competing interests.

## Authors' contributions

JZ, HL, ZD, and QZ designed and performed experiments. JZ and HL performed the statistical analysis and drafted the manuscript. DW and LL helped in drafting the manuscript and contributed specific information and critical analysis throughout the manuscript. All authors read and approved the final manuscript.
